# Short Communication: *In Vitro* Efficacy Testing of Praziquantel, Ivermectin, and Oxfendazole against *Taenia Solium* Cysts

**DOI:** 10.1155/2012/363276

**Published:** 2011-06-08

**Authors:** S. Cederberg, C. S. Sikasunge, Å. Andersson, M. V. Johansen

**Affiliations:** ^1^Department of Pharmacology and Pharmacotherapy, Faculty of Pharmaceutical Sciences, University of Copenhagen, Universitetsparken 2, 2100 Copenhagen, Denmark; ^2^Department of Paraclinical Studies, School of Veterinary Medicine, University of Zambia, P.O. Box 32379, Lusaka, Zambia; ^3^DBL-Centre for Health Research and Development, Department of Veterinary Pathobiology, Faculty of Life Sciences, University of Copenhagen, Thorvaldsensvej 57, 1870 Frederiksberg C, Denmark

## Abstract

Oxfendazole is recommended as the drug of choice for treating porcine cysticercosis. The drug does not kill brain cysts and is not registered for use in pigs. Latest its safety in the recommended dose has been questioned. The aim of this study was to investigate two alternative anthelminthics. The efficacy of praziquantel and ivermectin was compared to oxfendazole *In Vitro* on *Taenia solium*. Cysts of *T. solium* were isolated from infected pork and incubated in culture media together with the drugs. The degree of evagination was used as effect measurement and determined after 6 hours. Praziquantel had a half maximal effective concentration (EC_50_) of value 0.006 ± 0.001 **μ**g/mL. Ivermectin did not show any impact on the evagination in concentrations from 0.001 to 30 **μ**g/mL and neither did oxfendazole in concentrations from 0.001 to 50 **μ**g/mL.

## 1. Introduction

This is a short communication concerning porcine cysticercosis, an infectious disease caused by the larval stage of *Taenia solium. *This parasite is a zoonotic tapeworm transmitted between humans and between humans and pigs. Cysticercosis is widespread in Latin America, Asia, and Africa, and due to the link between human neurocysticercosis and epilepsy, control of this disease is now receiving increasing attention [1]. Single intervention is not an option against a parasitic infection, which is maintained in areas with no knowledge about the disease, poor sanitary conditions, free range management of pigs, and absence or inadequate meat inspection [2]. However, strategic use of effective anthelminthics in both humans and pigs is a realistic component of the short-term control of *T. solium. * Several drugs have been tested for porcine cysticercosis including flubendazole, albendazole, oxfendazole (OXF), and praziquantel (PZQ). OXF has shown efficacy to kill the cysts when tested in a single oral dose at 30 mg/kg [3–6]. Palomares et al. and Garcia-Dominguez et al. have likewise shown PZQ to be effective, when tested *in vitro* [7, 8], and a smaller efficacy is observed when administered in an oral formulation. The oral administration of OXF and PZQ is not suitable for free roaming pigs, due to uncertain dose in the pigs. Ivermectin (IVM) is a widely used anthelmintic in many cysticercosis endemic regions and is formulated as a subcutaneous (s.c.) drug, which is suitable for administration to free roaming pigs. The effect of IVM against cysticercosis has not previously been examined in a systematic study, but Perez-Serrano et al. have reported positive results of IVM treatment against human cysticercosis in a case study[9]. The objective of this study was to investigate the efficacy of the three anthelmintics PZQ, IVM, and OXF when tested *in vitro* against *T. solium* cysts.

## 2. Materials and Methods

Naturally infected pigs (8 to 24 months old) confirmed positive by tongue examination, were bought at Chibolya Small Animal Livestock Market in Lusaka, and transported to the School of Veterinary Medicine at the University of Zambia (UNZA), Lusaka, Zambia. At UNZA the pigs were slaughtered and the carcasses were cut into smaller pieces. Only cysts with fluid and an intact bladder wall were collected and washed in phosphate buffered saline (PBS) (Medicago, Sweden, article no. 09-9420-100). Up to 25 cysts were placed in each petri dish, and drug solution and culture media were added to yield a final volume at 20 mL, which was enough to cover 25 cysts. The culture medium consisted of 50% v/v bovine bile in isotonic sodium chloride (BDH Chemicals, England, product no. 30123). Bovine bile was collected from a slaughterhouse in Mazabuka at Turnpike Abattoir and stored at −20°C until use. 

PZQ (Dr. Ehrenstorfer, Lot nr. 80821, Germany) and IVM (Dr. Ehrenstorfer, Lot nr. 80715, Germany) were tested in final concentrations between 0.001 and 30 *μ*g/mL and OXF (Dr. Ehrenstorfer, Lot nr. 60505, Germany) was tested in final concentrations between 0.001 and 50 *μ*g/mL. All drugs were dissolved in 99.9% ethanol and further diluted in distilled water. The concentration range of ethanol in the final test samples was 0.0001–2.5%. Positive controls consisted of 20 mL culture media only and were incubated together with the samples each time. The cysts were incubated for 6 hours in a Yamato Incubator (Japan) at 37°C and subsequently investigated for evagination by visual inspection. Evagination had occurred, when the bladder wall opened and the neck and scolex emerged [10]. Evagination as effect measurement was established based on Sikasunge and coworkers [6]*.*

Prior to the *in vitro *drug experiments, storage control studies of the cysts were performed. Freshly isolated cysts from a pig were divided into four batches. The first batch was incubated just after slaughtering. The second, third, and fourth batches were stored at 4°C in 500 mL bottles, containing PBS and 100 cysts in each. After one, two, and three nights, respectively, the evagination of the batches was examined. Each batch contained 75 cysts equally distributed in 3 petri dishes. A solvent control study was additionally conducted, using ethanol at the following concentrations 0.5-1-2-3-4-5%. 75 cysts equally distributed in 3 petri dishes were used for each concentration in this experiment. 

The results from the storage control experiment and the solvent control experiment were analysed by One-Way ANOVA followed by Dunnett's multiple comparison test (*α* = 0.01). The observations were expressed as evaginated cyst in correlation with the positive control (in percentage). Observations from the three drug experiments were plotted against log to final drug concentrations. When a relationship was observed, the data were fitted to a sigmoidal dose-response curve (variable slope). All graphs and statistical analysis were made in GraphPad Prism 4.

## 3. Results

The storage experiment showed that there was no significant difference between the cysts ability to evaginate, when incubated right after slaughter or after one night storage at 4°C, whereas the evagination dropped significantly after 2 days (results not shown). Therefore cysts in this study were used right away or after one night of storage. The solvent control study showed no significant difference in the cysts ability to evaginate, when incubated in up 5% ethanol, but the viability of the evaginated cysts was significantly decreased at 2% ethanol (results not shown). 

**Figure 1:**
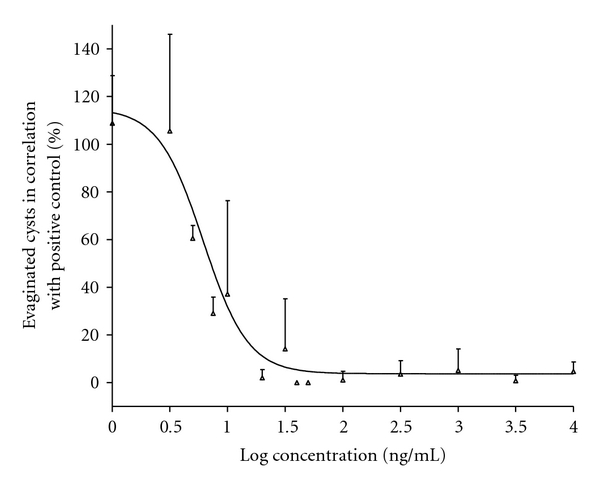
Percentage evaginated cysts in correlation with positive control, against the log concentration of praziquantel. The line is obtained by pooled data from four experiments and fitted to a dose-response sigmoidal curve (variable slope). The total number of cysts examined for this praziquantel study was 1803. The error bars represent standard deviation (S.D).

The efficacy of PZQ is shown in Figure 1. The graph shows approximately a mirror image of an s-shaped curve, when using log-transformed data at the x-axis and nonlog-transformed data at the y-axis, and a straight line is observed between 20 and 80% effects. The EC_50_ value was 0.006 *μ*g/mL ± 0.001 *μ*g/mL. 

**Figure 2:**
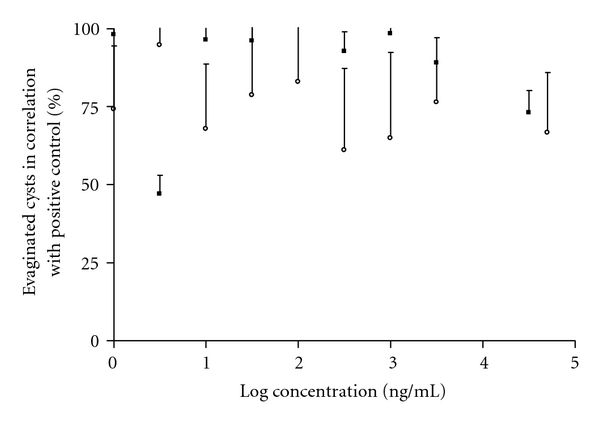
Percentage evaginated cysts in correlation whit positive control, against the log concentration of ivermectin and oxfendazole. The total number of cysts examined for the ivermectin study and the oxfendazole study was 750 for both drugs. The error bars represent standard deviation (S.D). ■: ivermectin; *⚪*: oxfendazole.

When testing IVM and OXF no significant differences from the positive controls were observed at any of the concentrations. The efficacy of IVM in concentrations between 0.001 and 30 *μ*g/mL and OXF in concentrations between 0.001 and 50 *μ*g/mL is presented in Figure 2. 

## 4. Discussion

PZQ had an EC_50_ value of 0.006 ± 0.001 *μ*g/mL on evagination of *T. solium* cysts *in vitro*. IVM and OXF did not show any impact on evagination from 0.001 to 30 *μ*g/mL (IVM) and 0.001 to 50 *μ*g/mL (OXF), which leaves PZQ as the only potent drug against *T. solium* cysts in this *in vitro* test system. The EC_50_ value of PZQ in this study is comparable to the data obtained by Garcia-Dominguez et al., which showed that PZQ inhibited evagination *in vitro* in the concentration range between 3·10^−4^ and 3·10^−3^ *μ*g/mL [8]. The PZQ graph in Figure 1 shows an S-shaped curve with an approximate strait line between 20 and 80% effect. This shape is comparable to the study by Palomares et al., who reported an EC_50_ value of 0.034 *μ*g/mL of PZQ *in vitro* after 6 hours of incubation [7]. This larger EC_50_ value could be due to the slightly different effect criterion set by Palomares et al., which is defined as total paralysis of the bladder wall and loss of cystic fluid [7]. A previous *in vivo* study likewise showed that PZQ has an impact on evagination, before the cysts oxygen consumption is inhibited, which indicates that PZQ influences evagination prior to cyst death [11]. 

Neither IVM nor OXF showed any effect on the evagination in the tested concentration area. Craven et al. have shown that after s.c. injection of 300 *μ*g/kg IVM in pigs, a maximal plasma concentration of 9.69 ng/mL occurred [12]. The dose 300 *μ*g/kg almost equals the recommended dose of IVM for pigs and the plasma level of 9.69 ng/mL is about 3.000 times lower than the highest tested concentration in this study. Hence it seems unnecessary to test IVM at larger concentrations. 

Although PZQ apparently is an effective drug against *T. solium* cysts, the practical administration of PZQ to pigs is still problematic. In Africa, PZQ is only commercially available in the form of 600 mg tablets, which is unsuitable for free roaming pigs. PZQ is available in a gel and pasta formulation in Europe, which would be a more suitable formulation. *In vivo* studies using an oral formulation of PZQ revealed that it was less effective than oral formulated OXF [13]. However, further studies testing different drug formulations should be encouraged. 

The observed results with OXF in our study fit poorly with results obtained *in vivo* [3–5]. In previous studies it is suggested that although anthelmintic drugs affect the parasite metabolism and damage the cysts, the death of the parasite occurs later as a result of direct attack from the host immune system [4, 14]. It has been demonstrated that it takes about one month before cysts viability is reduced to a level, which effectively controls the life cycle of *T. solium *[4, 6]. The theory is supported by this present study, where the lack of effect could be explained by the lack of a functional host immune system in the *in vitro *settings.

Our study has clearly demonstrated that PZQ but not OXF or IVM had a significant effect on *T. solium *cysts *in vitro,* but it also has shown that extrapolation from simple *in vitro *to *in vivo* drug efficacy studies is not possible. 

Even though no effect of IVM was observed in this study, the effect *in vivo* cannot be rejected. More work is needed on efficacy and effectiveness of anthelminthics against *T. solium* in pigs in order to come forward with evidence-based control recommendations.

##  Conflict of Interest

None of the authors have any conflict of interest in relation to the described work.
